# Heuristics as Bayesian inference under extreme priors

**DOI:** 10.1016/j.cogpsych.2017.11.006

**Published:** 2018-05

**Authors:** Paula Parpart, Matt Jones, Bradley C. Love

**Affiliations:** a University College London, United Kingdom; b University of Colorado Boulder, United States; c The Alan Turing Institute, United Kingdom

**Keywords:** Heuristics, Bayesian inference, Decision making, Ridge regression

## Abstract

Simple heuristics are often regarded as tractable decision strategies because they ignore a great deal of information in the input data. One puzzle is why heuristics can outperform *full-information* models, such as linear regression, which make full use of the available information. These “less-is-more” effects, in which a relatively simpler model outperforms a more complex model, are prevalent throughout cognitive science, and are frequently argued to demonstrate an inherent advantage of simplifying computation or ignoring information. In contrast, we show at the computational level (where algorithmic restrictions are set aside) that it is never optimal to discard information. Through a formal Bayesian analysis, we prove that popular heuristics, such as tallying and take-the-best, are formally equivalent to Bayesian inference under the limit of infinitely strong priors. Varying the strength of the prior yields a continuum of Bayesian models with the heuristics at one end and ordinary regression at the other. Critically, intermediate models perform better across all our simulations, suggesting that down-weighting information with the appropriate prior is preferable to entirely ignoring it. Rather than because of their simplicity, our analyses suggest heuristics perform well because they implement strong priors that approximate the actual structure of the environment. We end by considering how new heuristics could be derived by infinitely strengthening the priors of other Bayesian models. These formal results have implications for work in psychology, machine learning and economics.

## 1 Introduction

**Fig. 1:**
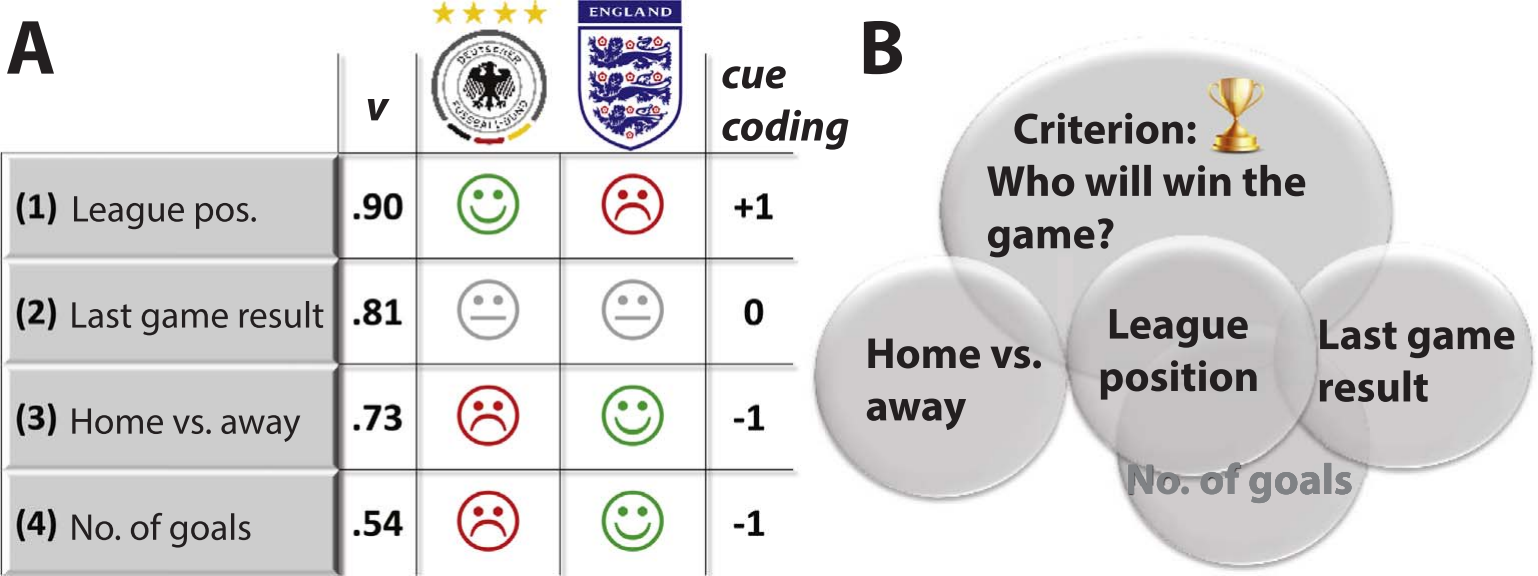
Illustrative example of a binary prediction task. (A) Predicting whether Team Germany or England will win is based on four cues: league position, last game result, home vs. away match, and recent goal scoring. Cue validities (*v*) reflect the relative frequency with which each cue makes correct inferences across many team comparisons (formula in Appendix A). Smiley and frowning faces indicate which team is superior on each cue, whereas a grey face indicates the two teams are equal on that cue. For modeling, a cue is coded +1 when it favors the team on the left (Germany), −1 when it favors the team on the right (England), and 0 when the teams are equal along that cue. (B) Irrespective of cue validity, cues can co-vary (illustrated by overlap) with the criterion variable but also with each other. The heuristics considered here ignore this covariance among cues.

Many real-world prediction problems involve binary classification based on available information, such as predicting whether Germany or England will win a soccer match based on the teams’ statistics. A relatively simple decision procedure would use a rule to combine available information (i.e., *cues*), such as the teams’ league position, the result of the last game between Germany and England, which team has scored more goals recently, and which team is home versus away. One such decision procedure, the *tallying heuristic*, simply checks which team is better on each cue and chooses the team that has more cues in its favor, ignoring any possible differences among cues in magnitude or predictive value (Czerlinski et al., 1999, Dawes, 1979). In the scenario depicted in Fig. 1A this heuristic would choose England. Another algorithm, *take-the-best* (TTB), would base the decision on the best single cue that differentiates the two options. TTB works by ranking the cues according to their *cue validity* (i.e., predictive value), then sequentially proceeding from the most valid to least valid until a cue is found that favors one team over the other (Gigerenzer & Goldstein, 1996). Thus TTB terminates at the first discriminative cue, discarding all remaining cues.

In contrast to these heuristic algorithms, a *full-information model* such as linear regression would make use of all the cues, their magnitudes, their predictive values, and observed covariation among them. For example, league position and number of goals scored are highly correlated, and this correlation influences the weights obtained from a regression model (Fig. 1B). Although such covariances naturally arise and can be meaningful, the cue validities used by the tallying and TTB heuristics completely ignore them (Martignon & Hoffrage, 1999). Instead, cue validities assess only the probability with which a single cue can identify the correct alternative, as the proportion of correct inferences made by that cue alone across a set of binary comparisons (formal definition in Appendix A). When two cues co-vary highly, they essentially provide the same information, but heuristics ignore this redundancy and treat the related cues as independent information sources. In the heuristic literature, the learner is usually assumed to learn cue validities from past experiences (i.e., the training data) (Gigerenzer and Goldstein, 1996, Gigerenzer and Todd, 1999).

Heuristics have a long history of study in cognitive science, where they are often viewed as more psychologically plausible than full-information models, because ignoring data makes the calculation easier and thus may be more compatible with inherent cognitive limitations (Bobadilla-Suarez and Love, 2018, Kahneman, 2003, Simon, 1990, Tversky and Kahneman, 1974). This view suggests that heuristics should underperform full-information models, with the loss in performance compensated by reduced computational cost. This prediction is challenged by observations of *less-is-more* effects, wherein heuristics sometimes outperform full-information models, such as linear regression, in real-world prediction tasks (Chater et al., 2003, Czerlinski et al., 1999, Dawes, 1979, Gigerenzer and Goldstein, 1996, Goldstein and Gigerenzer, 2002, Hogarth and Karelaia, 2007, Katsikopoulos et al., 2010). These findings have been used to argue that ignoring information can actually improve performance, even in the absence of processing limitations. For example, Gigerenzer and Todd (1999) write, “There is a point where too much information and too much information processing can hurt” (p. 21). Likewise, Gigerenzer and Brighton (2009) conclude, “A less-is-more effect, however, means that minds would not gain anything from relying on complex strategies, even if direct costs and opportunity costs were zero” (p. 111).

Less-is-more arguments also arise in other domains of cognitive science, such as in claims that learning is more successful when processing capacity is (at least initially) restricted (Elman, 1993, Newport, 1990). Contrary to existing claims, we argue there is no inherent computational advantage to simplicity of information processing. Less-is-more effects can arise only when the space of models under consideration is limited to a particular family or architecture. At a computational level of analysis, where restrictions on algorithms are set aside (Marr, 1982), more information is always better.

We cast our argument in a Bayesian framework, wherein additional information (input data) is always helpful but must be correctly combined with appropriate prior knowledge. We first prove that the tallying and TTB heuristics are equivalent to Bayesian inference under the limit of an infinitely strong prior. This connection suggests that heuristics perform well because their relative inflexibility amounts to a strong inductive bias, one that is suitable for many real-world learning and decision problems.

We then use this connection to define a continuum of Bayesian models, determined by parametric variation in the strength of the prior. At one end of the continuum (infinitely diffuse prior), the Bayesian model is equivalent to a variant of linear regression, and at the other end (infinitely strong prior) it is equivalent to a heuristic. Although the Bayesian models mimic the heuristics perfectly in the limit, a crucial difference is that the Bayesian account regulates cue weights but never discards any information. The models are tested on classic datasets that have been used to demonstrate superiority of the heuristics over linear regression, and in all cases we find that best performance comes from intermediate models on the continuum, which do not entirely ignore cue weights or cue covariance but that nonetheless down-weight this information via the influence of their priors. These results suggest that the success of heuristics, and findings of less-is-more effects more broadly in cognitive science, are due not to a computational advantage of simplicity per se, but rather to the fact that simpler models can approximate strong priors that are well-suited to the true structure of the environment.

## 2 Bias, variance, and Bayesian inference

The current explanation for less-is-more effects in the heuristics literature is based on the bias-variance dilemma (Gigerenzer & Todd, 1999). The present paper extends this Frequentist concept into a Bayesian framework that formally links heuristics and full-information models. From a statistical perspective, every model, including heuristics, has an inductive *bias*, which makes it best-suited to certain learning problems (Geman, Bienenstock, & Doursat, 1992). A model’s bias and the training data are responsible for what the model learns. In addition to differing in bias, models can also differ in how sensitive they are to sampling variability in the training data, which is reflected in the *variance* of the model’s parameters after training (i.e., across different training samples).

**Fig. 2:**
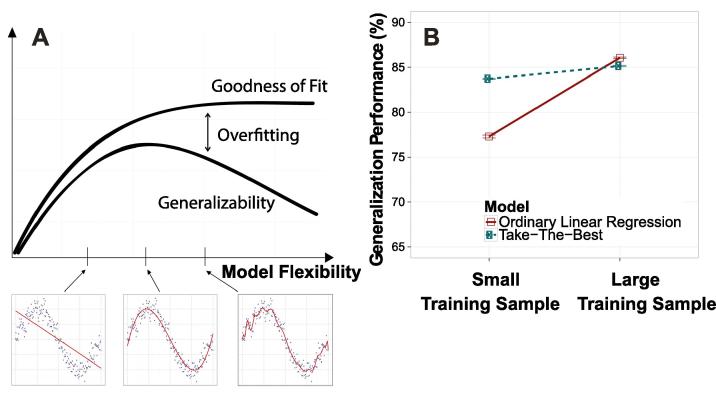
The concept of overfitting. (A) More flexible models can fit the training sample better (goodness of fit), accounting for most of the variability. However, these models can fare poorly in generalization tasks that test on novel samples (generalizability) (Pitt & Myung, 2002). (B) Our re-analysis of a dataset (Czerlinski et al., 1999) used to evaluate heuristics (predicting house prices) finds that TTB outperforms ordinary linear regression at generalization when the training sample is small (20 training cases). However, the pattern reverses when the training sample is enlarged (100 training cases). Error bars represent ±SEM. Details are in Appendix A.

A core tool in machine learning and psychology for evaluating the performance of learning models, *cross-validation*, assesses how well a model can apply what it has learned from past experiences (i.e., the training data) to novel test cases (Kohavi, 1995). From a psychological standpoint, a model’s cross-validation performance can be understood as its ability to generalize from past experience to guide future behavior. How well a model classifies test cases in cross-validation is jointly determined by its bias and variance. Higher flexibility can in fact hurt performance because it makes the model more sensitive to the idiosyncrasies of the training sample. This phenomenon, commonly referred to as *overfitting*, is characterized by high performance on experienced cases from the training sample but poor performance on novel test items. Overfitted models have high goodness of fit but low generalization performance (Fig. 2A; see Pitt & Myung, 2002).

Bias and variance tend to trade off with one another such that models with low bias suffer from high variance and vice versa (Geman et al., 1992). With small training samples, more flexible (i.e., less biased) models will overfit and can be bested by simpler (i.e., more biased) models such as heuristics. As the size of the training sample increases, variance becomes less influential and the advantage shifts to the complex models (Chater et al., 2003). Indeed, in a reanalysis of a dataset used to evaluate heuristics (Czerlinski et al., 1999), we find that the advantage for the heuristic over linear regression disappears when training sample size is increased (Fig. 2B).

The Bayesian framework offers a different perspective on the bias-variance dilemma. Provided a Bayesian model is correctly specified, it always integrates new data optimally, striking the perfect balance between prior and data. Thus using more information can only improve performance. From the Bayesian standpoint, a less-is-more effect can arise only if a model uses the data incorrectly, for example by weighting it too heavily relative to prior knowledge (e.g., with ordinary linear regression, where there effectively is no prior). In that case, the data might indeed increase estimation variance to the point that ignoring some of the information could improve performance. However, that can never be the best solution. One can always obtain superior predictive performance by using all of the information but tempering it with the appropriate prior. The results in the remainder of this paper demonstrate this conclusion explicitly.

## 3 Tallying as a limiting case of regularized regression

The first Bayesian model we develop is conceptually related to ridge regression (Hoerl & Kennard, 1970), a successful regularized regression approach in machine learning. Ridge regression extends ordinary linear regression by incorporating a penalty term that adjusts model flexibility to improve weight estimates and avoid overfitting (Fig. 2A). The types of tasks we model in this paper are binary comparisons, where each input represents a comparison between two alternatives on a set of cues, and the output represents which alternative has the greater value on some outcome variable. Consider a training set of input-output pairs $\left(x_{1}\text{,}y_{1}\right)\text{,}\ldots \text{,}\left(x_{n}\text{,}y_{n}\right)$ with $x_{i}\in {\left\{-1\text{,}0\text{,}1\right\}}^{m}$ and $y_{i}\in \{-1\text{,}1\}$. An example is Fig. 1A, where the explanatory variables (x) encode which soccer team is superior on each cue, and the outcome variable (*y*) indicates which team won each comparison (match). The aim in any linear regression problem is to estimate the weights, i.e., a vector of regression coefficients $w={\left[w_{1}\text{,}\ldots \text{,}w_{m}\right]}^{T}$, such that prediction error between *y* and Xw is minimized. The weights estimated by ridge regression are defined by

(1) w^ridge=argminwy-Xw2︸Goodness-of-Fit+θw2︸Penalty Term,

where the penalty parameter θ is nonnegative. ${\left\|\cdot \right\|}^{2}$ denotes the square of the Euclidean norm, $y={\left[y_{1}\text{,}\ldots \text{,}y_{n}\right]}^{T}$ is the outcome variable defined over all *n* binary comparisons in the training sample, and X is an n×m matrix with one column for each of the *m* predictor variables $x_{j}$. When the penalty parameter equals zero, ridge regression is concerned only with goodness of fit (i.e., minimizing squared error on the training set). For this special case, ridge regression is equivalent to ordinary linear regression, which is highly sensitive to sampling variability in the training set. As the penalty parameter increases, the pressure to shrink the weights increases, reducing them to zero as θ→∞. Thus larger values of θ lead to stronger inductive bias, which can reduce overfitting by reducing sensitivity to noise in the training sample. However, the optimal setting of θ will always depend on the environment from which the weights, cues, and outcomes were sampled.

**Fig. A2:**
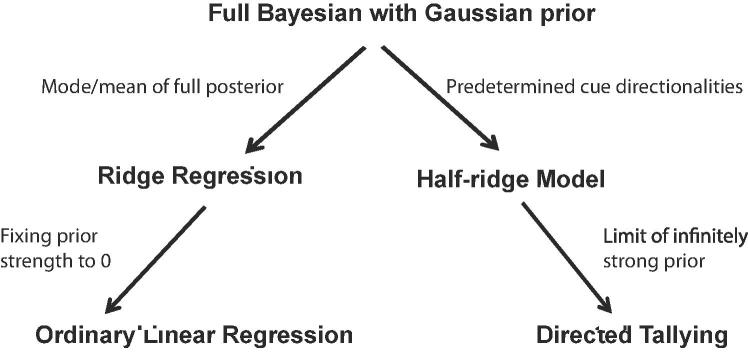
Formal relationships among full Bayesian regression, ridge regression, ordinary least-squares linear regression, the Bayesian half-ridge model, and the directed tallying heuristic. The lower-right arrow represents the main contribution of this paper, that a heuristic is a limiting case of Bayesian inference (here, the half-ridge model) with an infinitely strong prior.

The ridge penalty term is mathematically equivalent to a Gaussian Bayesian prior on the weights, where θ is inversely proportional to the prior variance $\eta^{2}$ of each $w_{i}$ (i.e., $\theta =\sigma^{2}/\eta^{2}$, where $\sigma^{2}$ is the variance of the error in *y*, also assumed to be Gaussian). In the Bayesian interpretation, the strength of the prior is thus reflected by $1/\eta^{2}$, growing stronger as η→0. This prior distribution is combined with observations from the training sample to form a posterior distribution (also Gaussian) over the weights. Like ordinary linear regression, ridge regression provides a point estimate for the weights, equal to the mean (and also the mode) of the full Bayesian posterior distribution (Marquaridt, 1970, Ripley, 2007). The conceptual relationships among ridge regression, ordinary linear regression, and the Bayesian model are illustrated in Appendix A, Fig. A2.

### 3.1 Half-ridge model and tallying

Our Bayesian derivation of the tallying heuristic extends ridge regression by assuming the directionalities of the cues (i.e., the signs of the true weights) are known in advance. For example, being higher in the league standings will, if anything, make a team more likely (not less) to win a given match. This assumption is concordant with how the tallying heuristic was originally proposed in the literature (Dawes, 1979). We refer to this definition of the tallying heuristic as *directed tallying* in order to differentiate it from the version of the tallying heuristic that learns cue directionalities from the training data (Czerlinski et al., 1999). Thus we define the prior for each weight as half-Gaussian, truncated at zero (right-hand side in Fig. A2, Appendix A), and we refer to this Bayesian model as the *half-ridge* model. Formally, the joint prior is defined by

(2) $$w∼N{\left(0\text{,}\Sigma \right)}_{|w\in O}\text{,}$$

where $\Sigma =\eta^{2}I\text{,}$ is the covariance matrix among the weights (prior to truncation) and $\eta^{2}$ determines the variance for each weight. The restriction notation, |w∈O, indicates we truncate the distribution to one orthant $O\subset R^{m}$, defined by the predetermined directionalities of the cues. For example, if the cues were assumed all to have positive (or null) effects on the outcome, then O would equal $\left\{w\in R^{m}|\text{∀}i\text{,}w_{i}⩾0\right\}$. Under this assumption, the posterior distribution inherits the same truncation (see Appendix A for derivations). The important question is what happens to this posterior as the prior becomes arbitrarily strong, that is, as η→0. Just as with increasing the penalty parameter in regular ridge regression, strengthening the prior in the half-ridge model shrinks the posterior weights toward zero (Eq. (3)). However, the ratios of the weights—that is, the relative inferred strengths of the cues—all converge to unity. This result can be seen through a simple rescaling of the weights, which has no impact on a binary comparison task. In particular, we show in the Appendix A that the posterior distribution for w/η obeys

(3) $$\frac{w}{\eta }\overset{d}{\to }N{\left(0\text{,}I\right)}_{|w\in O}\;\text{as}\;\eta \to 0$$

conditional on X and y (where $\overset{d}{\to }$ indicates convergence in distribution). Consequently, the rescaled weights all have the same posterior mean in the limit:

(4) $$\underset{\eta \to 0}{\lim}\;E\left[\left(\frac{w_{i}}{\eta }\right|X\text{,}y\right]=\pm \sqrt{\frac{2}{\pi }}\text{,}$$

with signs determined by each cue’s assumed directionality. Therefore, the optimal decision-making strategy under the Bayesian half-ridge model converges to a simple summation of the predictors—that is, the directed tallying heuristic. Note that, under this limit, the model becomes completely invariant to the training data. In particular, it ignores how strongly each cue is associated with the outcome in the training set (i.e., magnitudes of cue validities). At the other extreme, as the prior becomes extremely weak (η→∞), the Bayesian half-ridge model converges to a full regression model akin to ordinary linear regression in that it differentially weights the cues (e.g., more predictive cues receive higher weights than less predictive cues), the only difference being that the weights are constrained to have their predetermined signs. In conclusion, the half-ridge model demonstrates how the directed tallying heuristic arises as an extreme case of a Bayesian prior on the distributions of weights in the environment, and it shows that tallying and linear regression can be related by a continuum of models that differ only in the strength of this prior.

### 3.2 Heuristics vs. intermediate models

From a Bayesian perspective, the model that fares best on a given decision task should be the one with a prior most closely matching the data’s generating process. In many decision environments, cues differ in their predictiveness, but these differences are not arbitrarily large (i.e., the cue weights are not drawn uniformly from all real numbers). An advantage of the Bayesian half-ridge framework is that it specifies a continuum of models between the extremes of linear regression and the directed tallying heuristic. For many environments, the best-performing model should lie somewhere between these two extremes. Furthermore, the best-performing model should not change with different training set sizes (cf. Fig. 2), because—unlike the Frequentist phenomenon of bias-variance tradeoff—a correctly specified Bayesian model is guaranteed to find the optimal tradeoff between prior and likelihood, for any sample size.

**Fig. 3:**
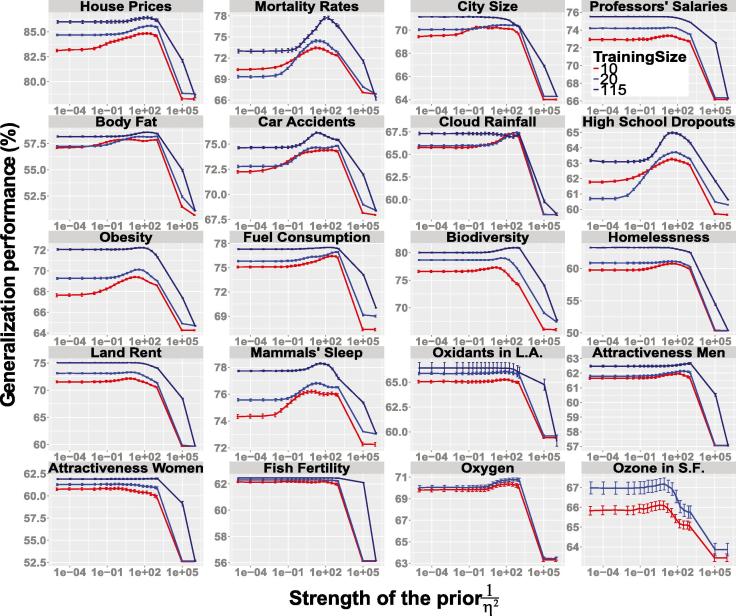
Generalization performance of the Bayesian half-ridge model by training sample size and as a function of the strength of the prior for 20 datasets for which heuristics have been previously evaluated (Czerlinski et al., 1999). The abscissa represents the strength of the prior, and the ordinate represents the predictive accuracy of the model on test comparisons. Note that an approximately infinitely strong prior on the far right of each graph (small values of η) corresponds to the directed tallying heuristic. Intermediate models (i.e., with a medium-strength prior) performed best in all datasets regardless of training sample size. Error bars represent ±SEM. Because the Oxygen and Ozone datasets contain less than 115 object pairs in total, training size 115 is not included for them. See Appendix A for details.

The Bayesian half-ridge model was simulated on 20 datasets that have been used to compare heuristic and regression approaches (Czerlinski et al., 1999). The key finding is that intermediate models perform best in all cases for all training sample sizes (see Fig. 3). Interestingly, the ordinary regression model (i.e., the limit of η→∞) outperforms the tallying heuristic (i.e., the limit of η→0). This discrepancy from past less-is-more results arises because cue directions are not learned in these simulations, and therefore there is no opportunity for the more flexible regression model to misestimate the cue directions. We do demonstrate less-is-more results in the 20 datasets (Czerlinski et al., 1999, Katsikopoulos et al., 2010) when comparing heuristics and regression models that estimate cue directions from the training set (Appendix A, Fig. A5, Fig. A6). The main finding, that intermediate half-ridge models outperform tallying in all 20 datasets, suggests that ignoring information is never the best solution. The best-performing model uses all the information in the training data, combining it with the appropriate prior.

## 4 A covariance-based Bayesian model and heuristics

In this section, we consider a second Bayesian model that, unlike the half-ridge model, learns cue directions from the training set and provides a unification of TTB, tallying, and linear regression. Given that ridge regression (L2 regularization) yields tallying, one might wonder whether a strong prior of a different functional form might yield the TTB heuristic. In particular, *lasso regression* (L1 regularization) (Ripley, 2007) is known to produce sparsity in cue selection (i.e., many weights are estimated as zero), and thus might be expected to yield TTB in the limit. Instead, derivations show that lasso regression also converges to tallying in the limit when the cue directionalities are known a priori. This result highlights the robustness of the conclusions of the previous sections, with tallying arising as a limiting case of Bayesian inference under a variety of different priors.

**Fig. 4:**
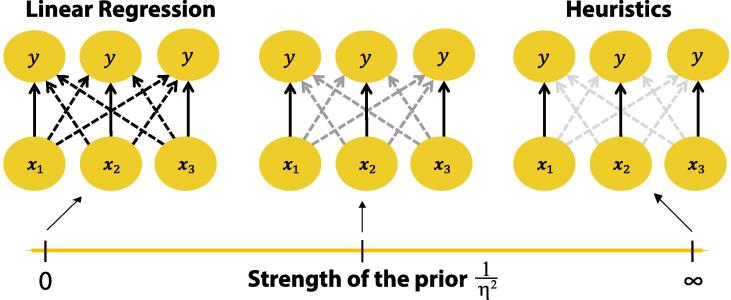
The prior of the COR model influences the posterior solution (i.e., the mean of the posterior on W) such that the model encompasses linear regression and the heuristics as extreme cases. In this example, there are m=3 cues, represented as vectors $x_{1}\text{,}x_{2}\text{,}x_{3}$, where one set of entries $x_{i1}\text{,}x_{i2}\text{,}x_{i3}$ pertains to the *i*th binary comparison. In order to establish a continuum of covariation sensitivity, the criterion variable is multiplexed as many times as there are cues (i.e., *m* times). The result is a multivariate regression problem with a dependent matrix Y of *m* columns of identical criterion variables. We refer to the dashed arrows as *cross-weights*, and the solid arrows as *direct weights*, corresponding respectively to the off-diagonal and diagonal entries of the weight matrix W. In an ordinary linear regression model, the estimated weights depend on the cue covariances. In contrast, a model structure without any of the cross-weights would revert to three simple regressions with exactly one predictor each ($x_{1}$, $x_{2}$, or $x_{3}$). Therefore, in the limit $1/\eta^{2}\to 0$, the prior does not penalize the cross-weights, and the set of mean posterior weights to each copy of the criterion variable is equal to the ordinary linear regression solution (leftmost network). At the other extreme, when $1/\eta^{2}\to \infty$, the cross-weights are shrunk to zero, and the knowledge captured in the direct weights becomes equivalent to that embodied by cue validities in heuristics that ignore covariation information (rightmost network). Between these two extreme values of $1/\eta^{2}$ lie models that are sensitive to covariation to varying degrees (middle network).

Given this formal result, we take a different approach. One key observation is that, unlike linear regression, both TTB and tallying rely on isolated cue-outcome relationships (i.e., cue validity) that disregard covariance information among cues. We use this insight to construct our second Bayesian model, with a prior that suppresses information about cue covariance but leaves information about cue validity unaffected. We refer to this model as Covariance Orthogonalizing Regularization (COR), because our regularization method essentially makes cues appear more orthogonal to each other. The strength of the prior yields a continuum of models (Fig. 4) defined by sensitivity to covariation among cues, which smoothly vary in their mean posterior weight estimates from those of ordinary linear regression to weights that are linear transforms of the heuristics’ cue validities (see Appendix A derivations).

In contrast to ridge regression, we express the regression problem in multivariate terms by multiplexing the outcome *m* times (the number of predictors), which allows the model to capture the sequential nature of TTB. As shown in Fig. 4, every copy of the output receives input from every cue, and thus the weights can be represented as an m×m weight matrix W. Unlike in ridge regression, where the Gaussian prior shrinks all model weights toward zero, only the cross-weights (i.e., the off-diagonal elements) are penalized. In the limiting case, when the precision of the prior, $1/\eta^{2}$, approaches ∞, the cross-weights reduce to zero and the posterior estimates for the direct (diagonal) weights are equivalent to cue validities as used by the heuristics (i.e., neglecting covariance information), up to a linear transformation. At the other extreme, when $1/\eta^{2}=0$, every copy of y has the same posterior for its set of weights, and the mean (and mode) of this posterior is equal to the ordinary linear regression solution. In particular, the covariance information is reflected in the posterior weights as it is in the ordinary regression solution.

The model weights are paired with a decision rule to classify test items. First, the vector $x_{i}=\left[x_{i1}\text{,}\ldots \text{,}x_{\mathrm{im}}\right]$ is multiplied by the mean posterior weight matrix $W^{*}$ to generate an output vector y^i=y^i1,…,y^im:

(5) y^i=xiW∗.

Note that using the posterior mean is equivalent to integrating over the full posterior distribution, due to the linearity of Eq. (5). The *TTB decision rule* is then applied to the resulting y^i as

(6) zi=y^i,ji∗whereji∗=argmaxjy^ij

and

(7) $${\text{choice}}_{i}=\left\{\begin{array}{cc} +1\;(\mathrm{left})\text{,} & \text{if}\;z_{i}>0\\ -1\;(\mathrm{right})\text{,} & \text{if}\;z_{i}<0\\ \;0\;(\mathrm{guess})\text{,} & \text{if}\;z_{i}=0\text{.} \end{array}\right)$$

**Fig. A3:**
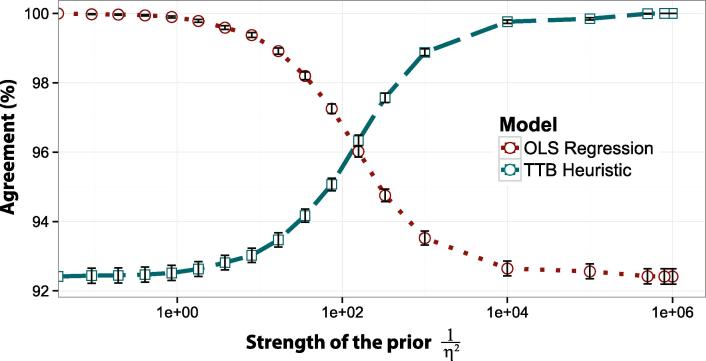
Agreement between the COR model (with TTB decision rule) and the TTB heuristic, as well as ordinary linear regression, as a function of the strength of the prior. As expected, agreement (i.e., proportion of equal predictions on test items) between the Bayesian COR model and TTB heuristic increased with a stronger prior, reaching an asymptote of perfect agreement as $1/\eta^{2}$ approached infinity. The opposite pattern held for ordinary linear regression, with agreement being perfect at $1/\eta^{2}=0$ and declining as the prior strength increases. Parallel results hold for the tallying decision rule (Fig. A4). The ordinate indicates the percentage agreement on test item choices in a simulated binary decision task with three cues. The simulated task contained 20 objects (e.g., an object represents a soccer team in Fig. 1), and the training set comprised all 190 possible pairwise comparisons of the objects. The test set represented all possible combinations of three cues which can take values of {−1, +1, 0} (coding scheme followed pattern in Fig. 1), and contained 26 cue combinations. The simulation process was repeated 1000 times, and error bars represent ±SEM across simulation runs. More details are provided in the Simulation 3 text.

Thus, the TTB decision rule selects the maximum absolute output (Eq. (6)) and takes the valence of that output as its choice (Eq. (7)). When $1/\eta^{2}\approx \infty$ (and the cross-weights are thus zero), the decision rule exhibits the exact sequential nature of the TTB heuristic, because then each output y^ij in Eq. (5) equals the value of the corresponding cue, $x_{\mathrm{ij}}$, times its cue validity. The largest output will correspond to the most valid cue that is not equal to zero (i.e., indifferent) for the particular test comparison. Thus, when the TTB decision rule is adopted, the COR model converges to the TTB heuristic as $1/\eta^{2}\to \infty$. In Appendix A, Fig. A3 shows simulations of an artificial binary prediction task similar to Fig. 1, demonstrating that the COR model (with TTB decision rule) and the TTB heuristic reach perfect agreement in their predictions as the prior becomes strong enough.

Notably, the tallying heuristic can also be derived from the COR model, in its undirected version that uses cue validities in the training data to infer cue directionalities. The *tallying decision rule* is defined by

(8) zi=∑jsigny^ij.

**Fig. A4:**
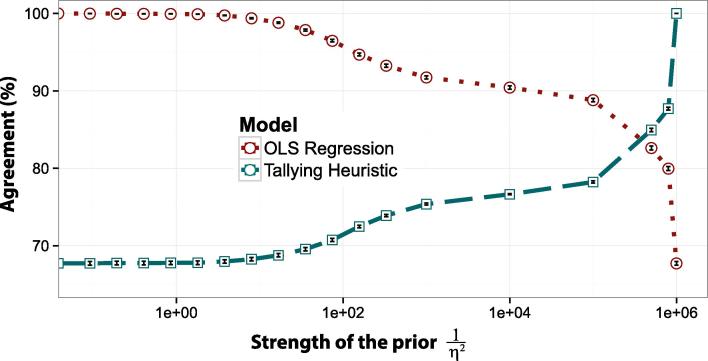
Agreement between the COR model (with tallying decision rule) and the tallying heuristic, as well as ordinary linear regression, as a function of the prior strength. The ordinate reflects the percentage agreement on test item choices in an artificial dataset. The artificial dataset used for this simulation is equivalent to the one displayed in Fig. A3 and described in the Simulation 2 text. Error bars represent ±SEM across simulation runs.

The tallying decision rule chooses the option with a majority of outputs in its favor (conveyed by their valences indicated by the sign function), irrespective of the magnitudes of the outputs. The choice is determined by Eq. (7), as in the TTB decision rule. When the tallying decision rule is adopted by the COR model, the model converges to the tallying heuristic in the limit as $1/\eta^{2}\to \infty$ (Fig. A4, Appendix A). Lastly, in the limit of $1/\eta^{2}\to 0$, either decision rule will yield decisions equivalent to ordinary linear regression. Under this limit, the outputs y^i produced according to Eq. (5) are all equal to the ordinary linear regression prediction (as outlined above), and both the TTB and tallying decision rules will yield a choice equal to the valence of that prediction.

**Fig. A5:**
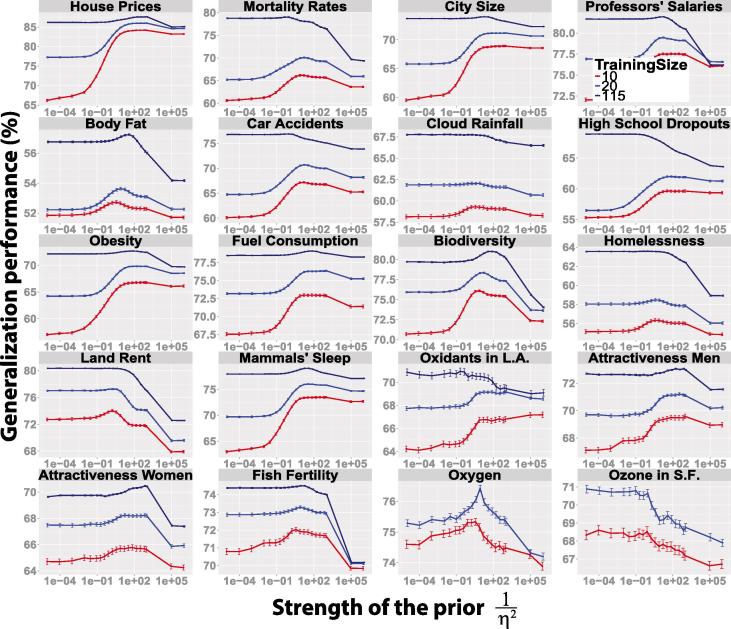
Generalization performance of the Bayesian COR model with the Tallying decision rule by training sample size in all 20 datasets that heuristics have been extensively tested on Czerlinski et al. (1999). The abscissa represents an increasing prior strength from left to right, and the ordinate represents the predictive accuracy of the model. Note that an approximately infinitely strong prior (e.g., $1/\eta^{2}$ = 1e+06) corresponds to the tallying heuristic, and a prior strength of zero ($1/\eta^{2}$ = 0) corresponds to ordinary linear regression. In 11 out of the 20 datasets, a less-is-more effect can be observed, where the tallying heuristic outperformed ordinary linear regression, with 10 and 20 training cases. For example, in the City Size, Car Accidents, and Mammals datasets, the tallying heuristic outperformed ordinary linear regression for training samples sizes of 10 or 20 training cases. However, the optimal performance could be found in the middle, i.e., for medium-strength priors. The optimal performance peak was robust across training sample sizes of 10, 20, and 115 training cases. In other datasets, such as Homelessness, Fish Fertility, and Women’s Attractiveness, ordinary linear regression outperformed tallying. However, the optimal performance for all datasets was found for intermediate COR models, i.e., for medium-strength priors. Error bars represent ±SEM.

**Fig. A6:**
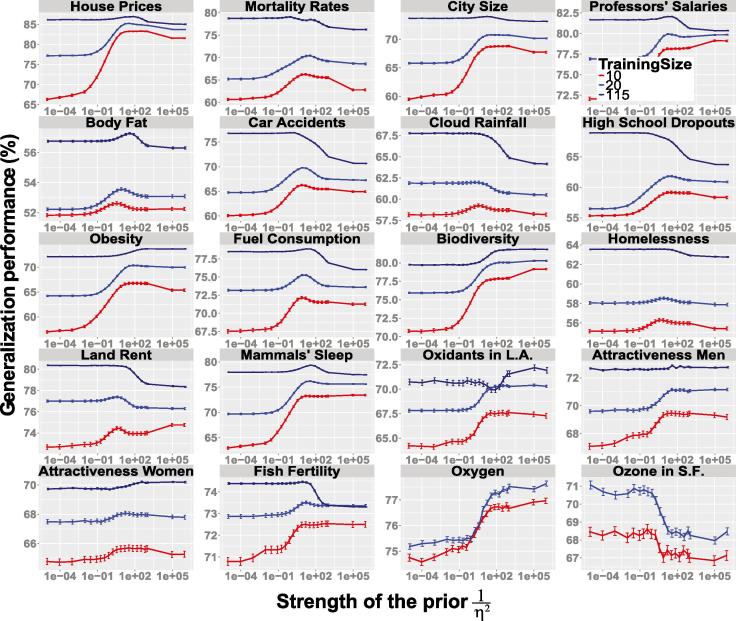
Generalization performance of the Bayesian COR model with the TTB decision rule by training sample size in all 20 datasets that heuristics have been extensively tested on Czerlinski et al. (1999). The abscissa represents an increasing prior strength from left to right, and the ordinate represents the predictive accuracy of the model. Note that an approx. infinitely strong prior (e.g., $1/\eta^{2}$ = 1e+06) corresponds to the TTB heuristic, and a prior strength of zero ($1/\eta^{2}$ = 0) corresponds to ordinary linear regression. In 18 out of the 20 datasets, a less-is-more effect can be observed, where the TTB heuristic outperformed ordinary linear regression, with 10 and 20 training cases. For example, in the House Prices, Mortality, City Size, and Professor Salaries datasets, the TTB heuristic outperformed ordinary linear regression for training samples sizes of 10 or 20, but the optimal performance could be found in the middle, i.e., for medium-strength priors. The optimal performance peak was roughly in the same place across training sample sizes of 10, 20, and 115 training cases. In other datasets, such as the Cloud Rainfall or the Ozone levels dataset, ordinary linear regression outperformed the TTB heuristic, but the optimal performance can still be found in the intermediate COR models, i.e., for medium-strength priors. Error bars represent ±SEM.

The COR model demonstrates how ordinary linear regression and both TTB and the tallying heuristic can be derived as extreme cases of a Bayesian prior defined by covariance expectation. Importantly, the only element varying across the continuum is the prior’s strength, and the prior is responsible for recovering the heuristics in the limit. The model converges to ordinary regression as the strength of the prior goes to zero regardless of the decision rule, and these model properties also hold under other forms of regularization (e.g. lasso regularization). As with the half-ridge model, we find that COR’s performance peaks for intermediate priors for all 20 datasets (Czerlinski et al., 1999) (Appendix A, Fig. A5, Fig. A6). Thus once again less is not more, as the heuristics are outperformed by a prior of finite strength that uses all information in the training data but nonetheless down-weights that information.

## 5 Discussion

A central message of this work is that, in contrast to less-is-more claims, ignoring information is rarely, if ever optimal (Gigerenzer and Brighton, 2009, Gigerenzer and Todd, 1999, Tsetsos et al., 2016). Heuristics may work well in practice because they correspond to infinitely strong priors that make them oblivious to aspects of the training data, but they will usually be outperformed by a prior of finite strength that leaves room for learning from experience (Fig. 3, and Fig. A5, Fig. A6 in Appendix A). That is, the strong form of less-is-more, that one can do better with heuristics by throwing out information rather than using it, is false. The optimal solution always uses all relevant information, but it combines that information with the appropriate prior. In contrast, no amount of data can overcome the heuristics’ inductive biases. The tallying heuristic is defined to entirely ignore differences in cue magnitude and predictiveness, unlike the intermediate half-ridge models, and cue validities are defined to entirely ignore covariation information, unlike the intermediate COR models.

Although the current contribution is formal in nature, it nevertheless has implications for psychology. In the psychological literature, heuristics have been repeatedly pitted against full-information algorithms (Chater et al., 2003, Czerlinski et al., 1999, Katsikopoulos et al., 2010) that differentially weight the available information or are sensitive to covariation among cues. The current work indicates that the best-performing model will usually lie between the extremes of ordinary linear regression and fast-and-frugal heuristics, i.e., at a prior of intermediate strength. Between these extremes lie a host of models with different sensitivity to cue-outcome correlations in the environment.

One question for future research is whether heuristics give an accurate characterization of psychological processing, or whether actual psychological processing is more akin to these more complex intermediate models. On the one hand, it could be that implementing the intermediate models is computationally intractable, and thus the brain uses heuristics because they efficiently approximate these more optimal models. This case would coincide with the view from the heuristics-and-biases tradition of heuristics as a tradeoff of accuracy for efficiency (Tversky & Kahneman, 1974). On the other hand, it could be that the brain has tractable means for implementing the intermediate models (i.e., for using all available information but down-weighting it appropriately). This case would be congruent with the view from ecological rationality where the brain’s inferential mechanisms are adapted to the statistical structure of the environment. However, this possibility suggests a reinterpretation of the empirical evidence used to support heuristics: heuristics might fit behavioral data well only because they closely mimic a more sophisticated strategy used by the mind.

Although we focused on explaining the success of two popular decision heuristics through a Bayesian analysis, our approach also suggests one could start with a Bayesian model and attempt to derive a novel heuristic by strengthening the prior. For example, in Gaussian process regression with a radial-basis kernel, the length-scale parameter determines how similar a training example needs to be to a test item to significantly influence the model’s prediction. Taking the limit as the length scale approaches zero might yield a heuristic akin to the nearest neighbor algorithm, in which the prediction is based solely on the most similar training item, ignoring all other training data. Such a solution would be algorithmically simple, but likely would be bested by models with intermediate prior strength. Whether this approach to deriving new heuristics would prove fruitful is an open question for future research.

There have been various recent approaches looking at the compatibility between psychologically plausible processes and probabilistic models of cognition (Bramley et al., 2017, Daw and Courville, 2008, Griffiths et al., 2015, Jones and Love, 2011, Lee and Cummins, 2004, Sanborn et al., 2010, Scheibehenne et al., 2013). These investigations are interlinked with our own, and while most of that work has focused on finding algorithms that approximate Bayesian models, we have taken the opposite approach. This contribution reiterates the importance of applying fundamental machine learning concepts to psychological findings (Gigerenzer & Brighton, 2009). In doing so, we provide a formal understanding of why heuristics can outperform full-information models by placing all models in a common probabilistic inference framework, where heuristics correspond to extreme priors that will usually be outperformed by intermediate models that use all available information.

## Figures and Tables

**Table A1:** Computation of cue validities: A binary prediction task where five object comparisons are made on the basis of three cues.

Comparison	Cue $x_{1}$	Cue $x_{2}$	Cue $x_{3}$	y	$r_{1}$	$w_{1}$	$x_{1}^{T}y$	$x_{1}^{T}x_{1}$
*1*	−1	−1	0	−1	1	0	1	1
*2*	1	−1	1	−1	0	1	−1	1
*3*	0	−1	0	1	0	0	0	0
*4*	1	1	1	1	1	0	1	1
*5*	1	1	−1	1	1	0	1	1
	$v_{1}=\frac{3}{4}$	$v_{2}=\frac{4}{5}$	$v_{3}=\frac{1}{3}$		$R_{1}=3$	$W_{1}=1$	$R_{1}-W_{1}=2$	$R_{1}+W_{1}=4$

The cue columns represent cue difference values, $x_{1}\text{,}x_{2}\text{,}x_{3}$ respectively, and are coded in the same way as the coding column in Fig. 1 in the main text. The criterion variable y contains the outcome of each comparison. $r_{1}$ and $w_{1}$ indicate whether cue $x_{1}$ predicted the outcome correctly or incorrectly (*r* = right, *w* = wrong) on each comparison, and $R_{1}$ and $W_{1}$ are the sums across all comparisons, $R_{1}=\sum r_{1}$ and $W_{1}=\sum w_{1}$. Then, the cue validity for cue $x_{1}$ is computed as $v_{1}=\frac{R_{1}}{W_{1}+R_{1}}$. The validities for $x_{2}$ and $x_{3}$ are defined similarly.

**Table A2:** Parameters of the simulation presented in Fig. 2B.

Parameter	Value
Number of objects	22
Number of pairwise comparisons	N=231
Number of cues	m=10
Class variable (which house had the higher actual sales price)	Binary, ±1
Absolute correlation between cues averaged over cue pairs	0.35
Sample cue validities	[1.00, 0.99, 0.94, 0.88, 0.83, 0.76, 0.73, 0.73, 0.72, 0.31]
Small training sample size	20 (∼9% of all pairwise comparisons)
Large training sample size	100 (∼43% of all pairwise comparisons)
Test sample size	N-20, N-100
Number of cross-validation repetitions	1000

**Table A3:** Parameters in the simulations presented in in Fig. 3.

Parameter	Value
Number of objects	11 to 395
Number of pairwise comparisons	N=55 to N=77,815
Number of cues	m=3 to m=18
Class variable (e.g., which house had the higher actual sales price)	Binary, ±1
Absolute correlation between cues averaged over cue pairs	Range = 0.12 to 0.63, mean = 0.31, median = 0.28, sd = 0.14
Training sample size	10, 20, 115
Test sample size	N-10, N-20, N-115
Number of cross-validation repetitions	1000
Error variance	$\sigma_{\varepsilon }^{2}=1$
Strength of prior	$1/\eta^{2}$ = [1000000, 100000, 1000, 700, 330.08, 156.81, 74.50, 35.39, 16.81, 7.99, 3.80, 1.80, 0.86, 0.41, 0.19, 0.09, 0.03, 0.01, 0.001, 0.0001, 0.00001]

**Fig. A1:**
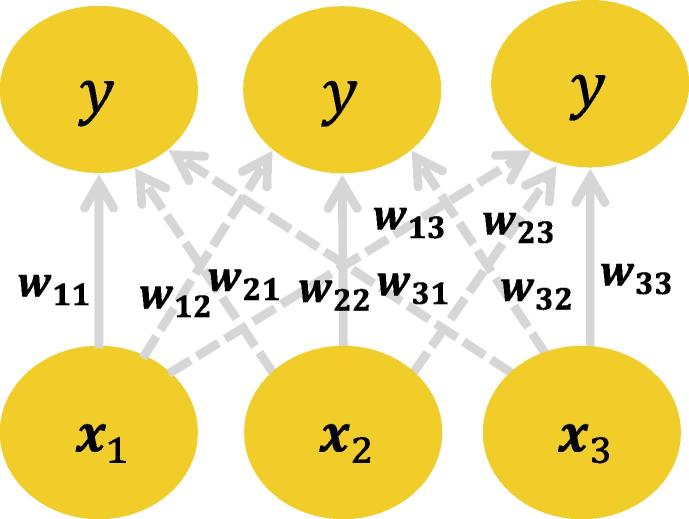
COR model architecture with m=3 cues as presented in Fig. 4 of the main paper. All y variables are replicas of one another and contain the same outcome information. Dashed arrows are called *cross-weights*, and solid arrows are called *direct weights*. Weight indices refer to the weight matrix W (Eq. (26)).

**Table A4:** Parameters of the artificial dataset presented in Figs. A3 and A4.

Parameter	Value
Number of objects	20
Number of pairwise comparisons	N=190
Number of cues	m=3
Class variable	Binary, ±1
Absolute correlation between cues averaged over cue pairs	0.26
Generating weights	Randomly sampled from an exponential distribution with rate parameter equal to 2
Training Sample Size	190
Test Sample Size	26
Number of cross-validation repetitions	1000
Error variance	$\sigma_{\varepsilon }^{2}=1$
Strength of prior	$1/\eta^{2}$ = [1000000, 800000, 500000, 100000, 10000, 1000, 700, 600, 500, 400, 330.08, 200, 156.81, 74.50, 35.39, 16.81, 7.99, 3.80, 1.80, 0.86, 0.41, 0.19, 0.09, 0]

**Table A5:** Parameters in the 20 datasets as presented in Fig. A5, Fig. A6

Parameter	Value
Number of objects	11 to 395
Number of pairwise comparisons	N=55 to N=77,815
Number of cues	m=3 to m=18
Class variable (e.g., which house had the higher actual sales price)	Binary, ±1
Absolute correlation between cues averaged over cue pairs	Range = 0.12 to 0.63, mean = 0.31, median = 0.28, sd = 0.14
Training sample size	10, 20, 115
Test sample size	N-10, N-20, N-115
Number of cross-validation repetitions	1000
Error variance	$\sigma_{\varepsilon }^{2}=1$
Strength of prior	$1/\eta^{2}$ = [1000000, 100000, 1000, 700, 330.08, 156.81, 74.50, 35.39, 16.81, 7.99, 3.80, 1.80, 0.86, 0.41, 0.19, 0.09, 0.03, 0.01, 0.001, 0.0001, 0.00001]

## Appendix A

### A.1 Cue validities

Fast and frugal heuristics, such as TTB and tallying, rely on cue validities for weights. Cue validities are defined for binary decision tasks, wherein two objects (e.g., two soccer teams) are compared on several cues and the inference is made about which object has the higher criterion value (i.e., which team will win the match). The criterion variable encodes the actual outcomes (e.g., which teams actually win the soccer matches), and can be coded as −1 and +1 as in Fig. 1 (main text). Cue validities, *v*, reflect the probability with which single cues can identify the correct alternative, and can be derived as the proportion of correct inferences made by each cue across a set binary comparisons (Martignon & Hoffrage, 1999):

(9) $$v=\frac{R}{R+W}$$

where *R* = number of correct predictions, *W* = number of incorrect predictions, and consequently, 0⩽v⩽1.

For example, Table A1 portrays a binary decision environment where five object comparisons are made on the basis of three cues. Note that the computation of cue validities ignores those cases where a cue predicts indifference between objects. A fundamental difference between cue validities and the regression weights derived by linear regression is that cue validities completely ignore covariance among cues. This is because cue validities are computed in isolation of one another, only considering how good each cue is at making correct inferences about the criterion separately from all other cues. In contrast, regression weights as estimated by a multiple linear regression model always consider the covariation among cues, as seen in the expression for the parameter estimate,

(10) w^=(XTX)-1XTy,

where $X^{T}X$ captures the covariances. In an ordinary linear regression analysis with multiple cues, the covariance among cues has a direct influence on the regression weights w^. If the regression weights were instead derived by regressing the criterion variable on each cue alone, i.e., eliminating all other cues from the model (single-predictor regression analysis), the weight magnitudes, valences as well rank order of weights would change. It can be shown that cue validities are a linear transformation of single-predictor regression weights (Martignon & Hoffrage, 1999), according to the following relationship:

(11) w^=2v-1.

This relation holds because, when there is a single predictor (x), the $X^{T}X$ term in Eq. (10) is equal to the number of cases where the predictor makes a prediction (x=±1), with cases where the predictor is indifferent (x=0) excluded. This can be seen from the computation in Table A1. That is,

(12) $$x^{T}x=R+W\text{.}$$

At the same time, $x^{T}y$ counts up all cases where a cue predicts the criterion (i.e., $x_{i}=y_{i}$) and subtracts those cases where the cue makes the opposite prediction (i.e., $x_{i}=-y_{i}$), while ignoring indifferent cases of $x_{i}=0$ (see Table A1). Thus

(13) $$x^{T}y=R-W\text{.}$$

Therefore, the single-predictor regression coefficient estimate w^ can be reformulated as

(14) w^=R-WR+W=2RR+W-R+WR+W=2v-1.

Note also that the expression $\frac{R-W}{R+W}$ in the first line of Eq. (14) represents the Goodman-Kruskal rank correlation (Martignon & Hoffrage, 1999). The linear relationship in Eq. (11) reveals that cue validities are a positive linear rescaling of single-predictor regression weights. Therefore they yield the same predictions in binary comparisons.

### A.2 Simulation 1: Reanalysis of a heuristic dataset (Fig. 2B)

Linear regression and the TTB heuristic were both fit to one of the original 20 datasets reported by the ABC Research Group (Czerlinski et al., 1999). In these original simulations (Czerlinski et al., 1999), the continuous values were transformed to binary values of 0 and 1 by median split. The criterion variable of the dataset analyzed in Fig. 2B encodes which of two houses has a higher actual sales price. There are 10 cues, which include things like the number of bedrooms, number of fireplaces, number of garage spaces, living space, current taxes, and the age of the house. We created all 231 possible pairwise comparisons of the original 22 houses. Both the linear regression model and TTB were cross-validated on the dataset by splitting the total number of pairwise comparisons randomly into training and test sets. The size of the training set was 20 comparisons (∼9% of all comparisons) or 100 comparisons (∼43% of all comparisons), and the test set was always the complementary set of comparisons. For each training set size, the cross-validation split into training and test sets was repeated 1000 times and performance of each model was averaged across these replications. Fig. 2B in the main text demonstrates the generalization performance, i.e., the out-of-sample performance, of both multiple linear regression and TTB as a function of the training set size (small or large). Error bars in Fig. 2B represent the variation in performance across all cross-validation splits, expressed as standard errors of the mean (see Table A2).

### A.3 Simulation 2: Generalization performance of the Bayesian half-ridge model in classic datasets (Fig. 3)

The goal of this simulation was to explore the predictive performance of the Bayesian half-ridge model in real-world datasets that have been previously used to evaluate heuristics on Czerlinski et al. (1999), and as a function of factors such as training sample size. The main text demonstrates the model’s performance in all original 20 heuristic datasets reported by the ABC Research Group (Czerlinski et al., 1999) (Fig. 3). These datasets span various domains from psychology to biology, health and environmental science, and range from predicting house prices and predicting mammals’ sleep time to predicting the attractiveness of famous men and women. The number of predictors varies from 3 (fish fertility dataset) to 18 (high school dropout dataset). In these classic datasets, the attributes are discretized at their medians into values of 0 and 1 from originally continuous data. For each dataset, we created all possible pairwise comparisons of the objects, with attributes coded as 0, 1 or −1 for each pair. The dependent variable was always binary and coded as −1 and +1.

The Bayesian half-ridge model was cross-validated on each dataset by splitting the total set of pairwise comparisons randomly into training and test sets. The size of the training set was varied between 10, 20 and 115 comparisons, and the test set was always the complementary set of comparisons. As two of the datasets, Oxygen and Ozone, only have 91 and 55 object pairs in total respectively, the large training sample size of 115 was excluded for those datasets. For each training sets size, the cross-validation split into training and test sets was repeated 1000 times and performance was averaged across all of these splits. Error bars in Fig. 3 represent the variation in performance across all 1000 cross-validation splits, expressed as standard errors of the mean. The half-ridge model predictions were derived by calculating the posterior weights from the training set using Eq. (21) below. The truncation used in Eq. (21) (i.e., the choice of orthant O) depended on the actual cue directions in the full dataset, following the assumption that the cue directions are known in advance. We derived a different posterior distribution for the weights under each value of the strength of the Bayesian prior (i.e., $1/\eta^{2}$ in Eq. (21)). Next, we used the mean of the posterior to make predictions for all comparisons in the test set. To assess the half-ridge model’s predictive accuracy on each test set, the predictions were compared to the actual binary criterion values in the test set, e.g., in the house price dataset this refers to which of two houses had the higher sales price. The model’s overall generalization performance was then computed as the average predictive accuracy across all 1000 test sets.

The performance results are depicted in Fig. 3 of the main text, which contains results for small, medium and large training sample sizes (10, 20 and 115 pairwise comparisons). The figure demonstrates the generalization performance of the half-ridge model for a range of $1/\eta^{2}$ = [1000000, 100000, 1000, 700, 330.08, 156.81, 74.50, 35.39, 16.81, 7.99, 3.80, 1.80, 0.86, 0.41, 0.19, 0.09, 0.03, 0.01, 0.001, 0.0001, 0.00001]. Importantly, an approximately infinite value of $1/\eta^{2}$ in the half-ridge model corresponds to the directed tallying heuristic (defined in the main text), as explained in the mathematical derivations below (Eqs. (24), (25)). Crucially, in the cross-validated evaluation of the half-ridge model, all models including heuristics and full regression are on the same level playing field. That is, our formulation places the heuristic within the same framework as other Bayesian models with the same prior, varying only in prior strength. The optimal prior strength (i.e., the best model within the continuum) may vary from one domain to another, but other than this choice of free parameter, the half-ridge model can be straightforwardly applied in any settings where the heuristic can.

We found that in all 20 datasets, the performance peaked for strengths of the prior lying between the two extremes of full regression (i.e., $1/\eta^{2}=0$) and the directed tallying heuristic (i.e., $1/\eta^{2}=\infty$). For all training set sizes, the directed tallying heuristic (as approximated by $1/\eta^{2}=1\text{,}000\text{,}000$) is outperformed by full regression, but intermediate models performed best. The reason that there are no less-is-more effects here (i.e., tallying outperforming regression) is that cue directions are not learned by the half-ridge model, as the assumption in the half-ridge model is that cue directions are known in advance (see mathematical derivations in Section A.6). Thus $1/\eta^{2}=0$ does not correspond to ordinary linear regression, but to a variant in which the weight estimates are constrained to have the correct signs (i.e., to lie in O). This means that there is less scope for the more flexible regression model to incorrectly estimate the true weights. In comparison, when we run ordinary regression (with unconstrained weights) on these datasets, we replicate the less-is-more effects previously found in these datasets (Czerlinski et al., 1999, Katsikopoulos et al., 2010), as can be seen in the COR model simulations of Fig. A5 (see Table A3).

In the current simulations, we defined training sets by sampling a subset of all possible comparisons (i.e., object pairs). In some past work, training sets have been defined by sampling a subset of the objects and then training on all pairs within the sampled subset. We have found both methods in the literature, i.e., sampling comparisons (Chater et al., 2003) and sampling objects (Czerlinski et al., 1999). To determine whether our results reported here would be dependent on this sampling decision, we compared both sampling methods. In short, the qualitative pattern of results is not dependent on the sampling method. When sampling objects rather than comparisons, we varied the training sample size between sampling 5, 7 and 16 objects, which correspond to 10, 21 and 120 possible comparisons for the training sets, respectively. We chose these training sample sizes to roughly match the training sample sizes used for the half-ridge simulations when sampling comparisons (i.e., 10, 20 and 115 training cases in Fig. 3). The pattern of results is almost the same under both sampling methods. The performance of models with extremely strong priors (i.e., directed tallying heuristic) is approximately the same under both methods (with some small error) and so is the performance of models with priors of zero strength (i.e., ordinary regression). Also, the location of the intermediate peak is approximately the same (with some small error) for both sampling methods in each of the 20 datasets.

### A.4 Simulation 3: Agreement between the Bayesian COR model and heuristics (Fig. A3, Fig. A4)

This simulation demonstrates how the COR model converges to the heuristics (i.e., tallying and TTB) as a function of the model’s prior strength in an artificial dataset. In order to generate the COR model’s predictions for artificial pairwise comparisons, the posterior weights (i.e., the model’s knowledge representations) were paired with either a TTB or a tallying decision rule. We simulated 1000 similar datasets overall and the model’s performance was averaged across all of them. Each artificial dataset was created as follows: The dataset had m=3 cues (e.g., cues in Fig. 1 would be rank, last game result, home vs. away match, and number of goals scored). We generated cue values on these three cues for 20 objects, by uniformly sampling cue values of 0 or 1. These cue values refer to the positive and negative smileys in the illustrative example of Fig. 1. An object refers to a single item (e.g., a soccer team in Fig. 1), not a pair of items to be compared. We then created all possible pairwise comparisons of the 20 objects, which results in 190 possible comparisons. A single pairwise comparison is like comparing two soccer teams, e.g., Team Germany versus Team England. For each pair, we computed the cue difference vector by subtracting the cue values of the second object from the first object. For example, in Fig. 1, the third column contains these cue difference values, which can take values of 1, −1 and 0. Next, we created a matrix of cue difference vectors with one row for each object pair. For each of the 1000 simulated datasets, we sampled m=3 weights from an exponential distribution with rate parameter equal to 2 as generating weights. Finally, we calculated a criterion variable by relying on the cue differences matrix, the generating weights, and additional Gaussian noise. The criterion variable contains the outcome for each object comparison, indicating which object won the comparison. If the cue-differences matrix is X, the vector of generating weights β, and the Gaussian noise ε, then the criterion variable y was generated through matrix multiplication,

(15) y=Xβ+ε.

The continuous y variable was thresholded at zero into +1 and −1 to indicate which object won the competition. Thus, y is coded in the same way as the cue differences above, with −1 indicating the second object won the competition and +1 indicating the first object won. All models, i.e., the COR model, the heuristics, and ordinary linear regression, were trained on this artificial dataset of 190 comparisons, and subsequently made predictions for a novel test set. The predictions on the test set were used to measure agreement among the different models. The test set was constructed according to a complete sampling approach where each possible combination of cue differences occurs once. For three cues with possible cue difference values of {−1, +1, 0}, there are 27 possible cue combinations. However, we deleted the test item that has zeros on all cue values, as it does not provide any information for discriminating among models (all models would guess for this comparison). Hence, the test matrix contains 26 test comparisons, one in each row. Each test pair corresponds to a novel pairwise comparison, e.g., between two soccer teams.

Linear regression was fit to the training set to estimate the ordinary least squares regression coefficients, and then cross-validated by predicting the 26 test items with the fitted optimal regression coefficients in matrix multiplication. The initial predictions are continuous and therefore were binarized by taking the signs of these predictions. Both the TTB and the tallying heuristic were fit to the artificial training set by estimating the cue validities according to Eq. (9). After learning the cue validities from the training sample, both heuristics made predictions with respect to the test pairs. The TTB heuristic makes predictions for each test pair by sequentially searching through cues in order of their validity until a first cue discriminates among the alternatives (i.e., its difference value is nonzero). The discriminating cue’s value (±1) is subsequently used for prediction. Tallying, in contrast, simply learns the signs of the cue validities, i.e., their unit weights (+1 when validity is greater 0.5, or −1 when validity is below 0.5), neglecting all validity magnitudes. At test, tallying then applies the unit weights to the unseen test pairs and counts up the positive and negative evidence for each alternative. The alternative with more evidence in its favor wins the comparison and is used for prediction. To derive the COR model predictions, we estimated the posterior weight matrix from the training set using the exact Bayesian posterior mean as detailed below in the Section A.7 (i.e., Eq. (30)). As we were interested in the change of the posterior weight matrix as a function of the strength of the prior in the model, we derived a different posterior estimate for each value of the strength of the prior. Next, we used the mean posterior weight matrix to make predictions with respect to the test set via matrix multiplication. If the cue differences for the test set are represented by a matrix M containing m=3 columns and 26 rows, and the mean posterior weight matrix $W^{*}$ is a 3×3 square matrix, then by matrix multiplication, the output is also a matrix Y with dimensions 26×3,

(16) $$Y={\mathrm{MW}}^{*}\text{.}$$

The output matrix Y contains the continuous predictions of the Bayesian model with respect to the three copies of y (see Section A.7). In order to convert this output matrix into the model’s choices, a TTB or a tallying decision rule was applied to each row of the output matrix as explained in the main text (Eqs. (6), (8)). Lastly, to measure convergence between the COR model (with the TTB or tallying decision rule) and the TTB heuristic or the tallying heuristic, we computed the agreement between models by dividing the number of equal predictions made on the test set by the total number of test comparisons. The agreement between the COR model and ordinary linear regression was computed in the same way. The simulation results of the COR model with the TTB decision rule are displayed in Fig. A3. The simulation results of the COR model with the tallying decision rule are displayed in Fig. A4.

The convergence findings were also verified by estimating the posterior mean through Markov chain Monte Carlo (MCMC) to sample from the true posterior probability distribution over the weight matrix W (see Table A4).

### A.5 Simulation 4: Generalization performance of the COR model in heuristic datasets (Fig. A5, Fig. A6)

The goal of this simulation was to explore the predictive performance of the COR model in real-world datasets, and as a function of factors such as training sample size. The supplementary figures, Fig. A5, Fig. A6, demonstrate simulations of the COR model on all original 20 heuristic datasets reported by the ABC Research Group (Czerlinski et al., 1999) that were also used to test performance of the half-ridge model in Fig. 3 of the main text, as described in simulation 2 of the SI above.

In these classic datasets, the attributes are discretized at their medians into 0 and 1 (from originally continuous data). We created all possible pairwise comparisons of the objects, which ends up in attribute data containing the possible values 0, 1 and −1. The dependent variable was always binary and coded as −1 or +1. The COR model was cross-validated on each dataset by splitting the total number of pairwise comparisons randomly into training and test set. The size of the training set was varied between 10, 20, and 115 comparisons, and the test set represented the complementary set of comparisons always. For each training set size, the cross-validation split into training and test set was repeated 1000 times and performance was averaged across all of them. Error bars in Fig. A5, Fig. A6 represent the variation in performance across all thousand cross-validation splits, expressed as standard errors of the mean.

The COR model predictions were derived by calculating the posterior weights based on the training set using the exact Bayesian posterior as in Eq. (30) below. That is, we could compute the posterior mean according to Eq. (30), by calculating the posterior weights for each copy of *y* one at a time. Next, we used the mean posterior weight matrix to make predictions with respect to the test set. We also validated these results with Markov chain Monte Carlo (MCMC), which samples directly from the Bayesian posterior over weight matrices. Since the Bayesian prior’s strength is represented by $1/\eta^{2}$, we derived a new posterior mean for each value of $1/\eta^{2}$. At the prediction stage, for each value of $1/\eta^{2}$, the mean posterior weight matrix was used to make predictions with respect to the test set via matrix multiplication (Eq. (16)), and was then combined with either of the two decision rules. To assess the COR model’s predictive accuracy, the predictions were compared to the actual criterion values in the test set, e.g., which of two houses had the higher sales price. When any of the models predicted a tie, i.e., a prediction of 0 which means the model is indeterminate about the binary outcome (label −1 or +1), the models were assumed to guess.

The performance results depicted in Fig. A5 represent the COR model with the tallying decision rule, while Fig. A6 displays the results for the TTB decision rule. The graphs in Fig. A5, Fig. A6 demonstrate the generalization performance of the COR model for different prior strengths $1/\eta^{2}$ = [0.00001, 0.0001, 0.001, 0.01, 0.03, 0.09, 0.19, 0.41, 0.86, 1.80, 3.80, 7.99, 16.81, 35.39, 74.50, 156.81, 330.08, 700, 1000, 1000000], under the condition of varying training set sizes, i.e., 10, 20 and 115 of the pairwise comparisons.

We found that, in 11 out of the 20 datasets, a less-is-more effect could be observed where the heuristic model, e.g., the tallying heuristic (Fig. A5) (or COR with infinitely strong prior), outperformed ordinary linear regression (prior strength of zero), with 10 and 20 training cases. However, the maximal performance was found for intermediate models, i.e., intermediate prior strengths, across all 20 datasets, and roughly in the same place across training sample sizes of 10, 20, and 115 training cases. This echoes a central finding from the half-ridge model (Fig. 3) where the performance peak could also be found in the middle, between the extremes of the heuristic and the full regression model. Results for the TTB decision rule were similar (Fig. A6), as the TTB heuristic (or COR with infinitely strong prior) outperformed ordinary linear regression (prior strength of zero), in 18 out of the 20 heuristic datasets with 10 and 20 training cases. Again, the performance peak could usually be found in the middle, i.e., for medium-strength priors (see Table A5).

As with the half-ridge simulations, the COR simulations reported here defined training sets by directly sampling pairs of objects (i.e., comparisons). We compared this approach to one of sampling objects (and training on all pairs in the sampled subset), to determine whether our results would be dependent on this sampling decision. In short, the qualitative pattern of results is not dependent on the sampling method. When sampling objects rather than comparisons, we varied the training sample size between sampling 5, 7, and 16 objects, which correspond to 10, 21, and 120 possible comparisons, respectively. We chose these training sample sizes to approximate the training sample sizes used for the COR simulations when sampling comparisons (i.e., 10, 20, and 115 training cases in Fig. A5, Fig. A6). For both the tallying and the TTB decision rule, the pattern is almost the same under both sampling methods. Performance of all models is lower overall by a few percent in accuracy when sampling objects, which makes sense as the models do not encounter test objects in the training set first. Additionally, models with weaker priors (i.e., closer to ordinary regression) showed a larger drop in performance under object sampling (especially for smaller training sizes) than did models with stronger priors (i.e., closer to the heuristics). Thus, sampling objects gives the heuristics a small advantage over ordinary regression for the training sample sizes considered here. However, the number of less-is-more effects (i.e., datasets in which heuristics outperform ordinary regression) is the same and they occur in the same environments for both sampling methods. Also, the location of the performance peak is the same (with some small error) under both sampling methods for both the TTB and tallying decision rules.

### A.6 Bayesian Half-ridge model

The half-ridge model’s prior is a truncated Normal distribution, equivalent to assuming the cue directions are known in advance (Dawes, 1979):

(17) $$w∼N{\left(0\text{,}\Sigma \right)}_{|w\in O}\text{,}$$

The restriction notation |w∈O indicates we truncate the distribution to one orthant $O\subset R^{m}$, defined by the predetermined directionalities of the cues, and renormalize. The covariance matrix Σ assumes the weights are all independent with variance $\eta^{2}$ (prior to truncation):

(18) $$\Sigma =\eta^{2}I\text{.}$$

Linear regression with an untruncated Gaussian prior (i.e., L2 regularization) yields a Gaussian posterior for the weights, having mean

(19) $${\left(X^{T}X+\sigma^{2}\Sigma^{-1}\right)}^{-1}X^{T}y$$

and variance

(20) $$\sigma^{2}{\left(X^{T}X+\sigma^{2}\Sigma^{-1}\right)}^{-1}\text{.}$$

The posterior for the half-ridge model inherits the truncation from the prior and is otherwise unchanged except for renormalization:

(21) $$w|X\text{,}y∼N{\left({\left(X^{T}X+\sigma^{2}\Sigma^{-1}\right)}^{-1}X^{T}y\text{,}\sigma^{2}{\left(X^{T}X+\sigma^{2}\Sigma^{-1}\right)}^{-1}\right)}_{|w\in O}\text{.}$$

To understand how the posterior behaves as the prior becomes arbitrarily strong, we can rescale the weights by 1/η and substitute Eq. (21) to rewrite the posterior as

(22) $$\left(\frac{w}{\eta }\right|X\text{,}y∼N{\left(\eta {\left(\eta^{2}X^{T}X+\sigma^{2}I\right)}^{-1}X^{T}y\text{,}{\left(\frac{\eta^{2}}{\sigma^{2}}X^{T}X+I\right)}^{-1}\right)}_{|w\in O}\text{.}$$

Rescaling the weights has no impact in a binary comparison task, so we can work with the distribution of w/η in place of that of w. The convenience of this rescaling is that the resulting distribution obeys a simple convergence:

(23) $$\frac{w}{\eta }\overset{d}{\to }N{\left(0\text{,}I\right)}_{|w\in O}\;\text{as}\;\eta \to 0$$

Therefore all weights converge to the same value, namely

(24) $$\underset{\eta \to 0}{\lim}E\left[\left(\frac{w_{i}}{\eta }\right|X\text{,}y\right]=\pm \sqrt{\frac{2}{\pi }}\text{,}$$

with signs determined by each cue’s assumed directionality. In particular, for any two weights *j* and *k*, their ratio converges to unity:

(25) $$\underset{\eta \to 0}{\lim}\frac{E\left[w_{j}|X\text{,}y\right]}{E\left[w_{k}|X\text{,}y\right]}=1\text{.}$$

Therefore the optimal decision-making strategy converges to a simple equal-weight strategy, or tallying strategy.

To understand this result intuitively, refer to Eqs. (20), (19) and note that the posterior mean and posterior variance both scale with the prior’s covariance matrix Σ as Σ approaches 0 (i.e., as the precision of the prior approaches ∞). Thus, the mean of each weight goes to 0 faster than its standard deviation, or in other words the coefficient of variation goes to 0. That fact is not consequential when the directions of the cues are unknown, but it is significant when the cue directions are known. In the latter case, the signs of w become the most important information the learner has, and in fact the training data become (in the limit η→0) irrelevant.

### A.7 Bayesian COR model

The COR model approach differs from standard regularized regression in that the prior modulates sensitivity for covariation among cues. This is achieved by expressing the regression problem in multivariate terms, by replicating the criterion variable y as many times as there are cues (i.e., *m* times). Due to this multiplexing, the model architecture implements *m* regression problems at once, meaning the criterion variable y is regressed onto all cues *m* times (Fig. A1). The weights constitute an m×m matrix W, with each column, $W_{\cdot j}$, representing the weights for the *j*th copy of the outcome, $y_{j}$:

(26) $$W=\left[\begin{array}{ccc} w_{11} & \cdots & w_{1m}\\ \vdots & \ddots & \vdots \\ w_{m1} & \cdots & w_{\mathrm{mm}} \end{array}\right]\text{.}$$

As in standard regression, the likelihood for each $y_{j}$ is given by a Gaussian with error variance $\sigma^{2}$:

(27) $$p(y_{j}|X\text{,}W)\propto \exp\left(-\frac{{\left({\mathrm{XW}}_{\cdot j}-y_{j}\right)}^{T}({\mathrm{XW}}_{\cdot j}-y_{j})}{2\sigma^{2}}\right)$$

where X is the matrix that contains the cue data and is indexed by trials and cues (i.e., n×m).

In contrast to ridge regression, where all weights are penalized equally, in the COR model only the off-diagonal elements of the weight matrix W are penalized, while the diagonal weights are left unpenalized. This is implemented by assuming an improper uniform prior on all $W_{\mathrm{ii}}$ (1⩽i⩽m) and a prior of $N\left(0\text{,}\eta^{2}\right)$ for all $W_{\mathrm{ij}}$ (i≠j). The joint distribution on W treats all weights as independent. The model architecture is illustrated in Fig. A1, were the solid arrows represent the diagonal weights (direct weights) and the dashed arrows represent the off-diagonal weights (cross-weights). Penalizing only the cross-weights has the effect that the strength of the prior ($1/\eta^{2}$) modulates the model’s sensitivity to covariation among cues. When $1/\eta^{2}=0$ (uniform prior on all weights), the posterior for the weights $W_{\cdot j}$ is identical for all $y_{j}$, with mean (and mode) equal to the ordinary least squares linear regression solution. As $1/\eta^{2}\to \infty$, the estimated cross-weights converge to zero, while the direct weights stay un-penalized. Thus in the limit the direct weight $W_{\mathrm{jj}}$ is the only nonzero weight in each column $W_{\cdot j}$. This means that each cue effectively has its own isolated regression (i.e., as if only direct weights were present in Fig. A1, with no cross-weights). These single-predictor regression weights are linear transforms of the cue validities as used by the heuristics (see proof in Eq. (14)). Therefore, in the limit, when the COR model weights are paired with a decision rule (Eq. 6 or 8), the model’s behavior converges to that of the respective heuristic.

To derive the posterior distribution for COR’s weight matrix, we observe first that the weights for the different copies of y are decoupled. More precisely, the prior, likelihood, and hence posterior all factor into separate functions, one for each set of weights $W_{\cdot j}$. Therefore we can derive the posterior separately for each set. The prior for each set of weights is given by

(28) $$p(W_{\cdot j})\propto \exp\left(-\frac{1}{2}W_{\cdot j}^{T}\Sigma W_{\cdot j}\right)$$

where Σ is the precision matrix, defined by $\Sigma_{\mathrm{jj}}=0\text{,}\;\Sigma_{\mathrm{ii}}=\frac{1}{\eta^{2}}$ for i≠j, and $\Sigma_{\mathrm{ik}}=0$ for i≠k. Combining this with the likelihood in Eq. (27) yields the posterior:

(29) $$\begin{array}{ccc} p(W_{\cdot j}|X\text{,}y) & \propto & \exp\left(-\frac{1}{2}W_{\cdot j}^{T}\left(\Sigma +\frac{1}{\sigma^{2}}X^{T}X\right)W_{\cdot j}+\frac{1}{\sigma^{2}}W_{\cdot j}^{T}X^{T}y\right)\\ & \propto & \exp\left(-\frac{1}{2\sigma^{2}}{\left(W_{\cdot j}-{\left(\Lambda +X^{T}X\right)}^{-1}X^{T}y\right)}^{T}\left(\Lambda +X^{T}X\right)\left(W_{\cdot j}-{\left(\Lambda +X^{T}X\right)}^{-1}X^{T}y\right)\right)\text{.} \end{array}$$

That is, the posterior for $W_{\cdot j}$ is a multivariate Gaussian with mean at

(30) $${\left(\Lambda +X^{T}X\right)}^{-1}X^{T}y$$

and covariance matrix equal to

(31) $$\sigma^{2}{\left(\Lambda +X^{T}X\right)}^{-1}\text{.}$$

The matrix Λ is interpretable as a matrix of penalties on the components of $W_{\cdot j}$, with $\Lambda_{\mathrm{jj}}=0$, $\Lambda_{\mathrm{ik}}=0$ for i≠k, and $\Lambda_{\mathrm{ij}}=\frac{\sigma^{2}}{\eta^{2}}$ for i≠j.

In the current work, this exact Bayesian solution of the COR model was used for deriving the model’s predictions in Simulation 3 (Fig. A3, Fig. A4) and Simulation 4 (Fig. A5, Fig. A6), by inserting the appropriate training data into Eq. (30) for the posterior mean weights. To derive the COR model’s predictions with respect to new test items, the posterior mean was multiplied with the test cue data to generate outputs (Eq. 5). Note that, by linearity, using the posterior mean gives the same result as integrating the prediction over the full posterior. These outputs were then combined with the tallying or TTB decision rule (Eq. (6) or (8)) to classify a test item. As detailed in the main text, the posterior weight matrix changes with the strength of the prior, $1/\eta^{2}$ in Λ in Eq. (30), and a different posterior weight matrix is estimated for each value of η.

Importantly, in contrast to the half-ridge model, the COR model is misspecified, because of the multiplexing of the criterion variable. The resulting model architecture is artificially multivariate despite the original prediction problem being univariate. Nevertheless, the COR model opens up new insights into the role of cue covariance in establishing a continuum between heuristics that rely on cue validity and full-information models. Penalizing only the cross-weights in the COR model architecture results in a regularization of covariance sensitivity in the model, with a continuum ranging from ordinary linear regression (fully sensitive to the covariance structure among cues) to heuristics that rely on cue validities (insensitive to any cue covariance).

A reviewer also suggested an alternative approach to building the model continua within a (Bayesian) logistic regression framework rather than the linear one used here. We believe this would be a useful avenue for further research, as part of a general program to build on the theoretical ideas introduced here to develop new, more powerful decision algorithms and to further link heuristics to other modeling approaches. For now, we note that the linear models we used here support the main conclusions just as well although they are not ideally tailored to the task being analyzed (i.e., where criterion values are binarized). The linear models were chosen in order to be consistent with past work (e.g., Czerlinski et al., 1999) to replicate less-is-more effects (e.g., the less-is-more findings in Fig. A5, Fig. A6) and in order to build a continuum between these models traditionally used in the heuristic literature. As with the comparison between half-ridge and COR, and the observation that different choices of regularization schemes (corresponding to Gaussian vs. Laplacian priors) lead to the same heuristics in the limit, we conjecture that heuristics can arise as limiting cases of many different Bayesian models that assume different generative processes.

Similarly, we chose the two particular heuristics, i.e., TTB and tallying, as they are among the most well-known fast-and-frugal heuristics, and because they are intuitive and arise in a number of contexts. Both heuristics have been repeatedly contrasted with “rational” full-information linear regression approaches (Czerlinski et al., 1999, Gigerenzer and Goldstein, 1996, Katsikopoulos et al., 2010), which makes them very suitable for consideration as part of a Bayesian inference model for our purpose. However, including other heuristics will provide a great extension of the current Bayesian program to better understand heuristics and their relationship to full-information models and Bayesian inference models. Fortunately, analyses with the initial two heuristics proved tractable, providing an opportunity to argue that less is not more when less involves ignoring (rather than down-weighting) information.
